# The DNA-binding protein CST associates with the cohesin complex and promotes chromosome cohesion

**DOI:** 10.1016/j.jbc.2021.101026

**Published:** 2021-07-30

**Authors:** P. Logan Schuck, Lauren E. Ball, Jason A. Stewart

**Affiliations:** 1 Department of Biological Sciences, University of South Carolina, Columbia, South Carolina, USA; 2 Department of Cell and Molecular Pharmacology, Medical University of South Carolina, Charleston, South Carolina, USA

**Keywords:** chromosome cohesion, cohesin, CST, DNA replication, CTC1, STN1, TEN1, fork stalling, Ac-SMC3, acetylation of SMC3, APH, aphidicolin, co-IP, coimmunoprecipitation, CPT, camptothecin, CST, CTC1-STN1-TEN1, HU, hydroxyurea, PLA, proximity ligation assay, SCC, sister chromatid cohesion, shSTN1, shRNA knockdown of STN1

## Abstract

Sister chromatid cohesion (SCC), the pairing of sister chromatids after DNA replication until mitosis, is established by loading of the cohesin complex on newly replicated chromatids. Cohesin must then be maintained until mitosis to prevent segregation defects and aneuploidy. However, how SCC is established and maintained until mitosis remains incompletely understood, and emerging evidence suggests that replication stress may lead to premature SCC loss. Here, we report that the ssDNA-binding protein CTC1-STN1-TEN1 (CST) aids in SCC. CST primarily functions in telomere length regulation but also has known roles in replication restart and DNA repair. After depletion of CST subunits, we observed an increase in the complete loss of SCC. In addition, we determined that CST associates with the cohesin complex. Unexpectedly, we did not find evidence of altered cohesin loading or mitotic progression in the absence of CST; however, we did find that treatment with various replication inhibitors increased the association between CST and cohesin. Because replication stress was recently shown to induce SCC loss, we hypothesized that CST may be required to maintain or remodel SCC after DNA replication fork stalling. In agreement with this idea, SCC loss was greatly increased in CST-depleted cells after exogenous replication stress. Based on our findings, we propose that CST aids in the maintenance of SCC at stalled replication forks to prevent premature cohesion loss.

As DNA is replicated, the sister chromatids must be held together until mitosis to ensure chromosomes are properly segregated between daughter cells. This process, known as sister chromatid cohesion (SCC), is facilitated by the cohesin complex (1, 2). In mammals, the cohesin complex is composed of a ring-like structure that encircles the DNA. The core structure is composed of SMC3, SMC1A, SCC1/RAD21, and SA1 or SA2. Cohesin loading and removal is tightly regulated during the cell cycle (3, 4, 5). In G1 phase and early S phase, cohesin is loaded onto chromatin but not stably bound. As the DNA is replicated, cohesin is passaged to the replicated sister chromatids and becomes stably bound until mitosis when it is removed to allow segregation of the chromatids into daughter cells. In addition to its essential role in SCC, the cohesin complex is involved in organizing topologically associated domains for cellular processes such as DNA repair and gene expression (6). Moreover, several recent studies found that replication stress causes perturbation in cohesin dynamics at stalled replication forks and premature SCC loss in human cells (7, 8, 9).

In this study, we present unexpected findings that human CTC1-STN1-TEN1 (CST) helps maintain SCC. CST is an replication protein A (RPA)-like, ssDNA-binding protein that is conserved from yeast to humans (10). CST primarily functions in telomere length regulation; however, it has also been shown to function in DNA replication and repair (11, 12). Although its role in DNA replication is still not well understood, it is proposed to aid in the replication of G-rich DNA sequences, such as telomeres, promote dormant origin firing, and negatively regulate origin licensing (13, 14, 15, 16). CST also interacts with several components of the replication machinery, including DNA polymerase α-primase, the MCM2-7 helicase, and AND-1/Ctf4 (14, 17, 18). Together, these findings provide strong evidence that CST is involved in DNA replication, presumably as a specialized *versus* general replication factor.

Here, we report that depletion of CST leads to premature SCC loss. Furthermore, we show that CST associates with the cohesin complex, suggesting that CST may directly influence SCC. Upon further investigation, we found that the association between cohesin and CST is increased after replication stress and CST prevents SCC loss after treatment with several replication inhibitors. Together, our findings identify CST as a new factor involved in preventing premature cohesion loss and suggest that it does so by stabilizing cohesion after replication fork stalling.

## Results

### Depletion of CST results in SCC loss

**Figure 1:**
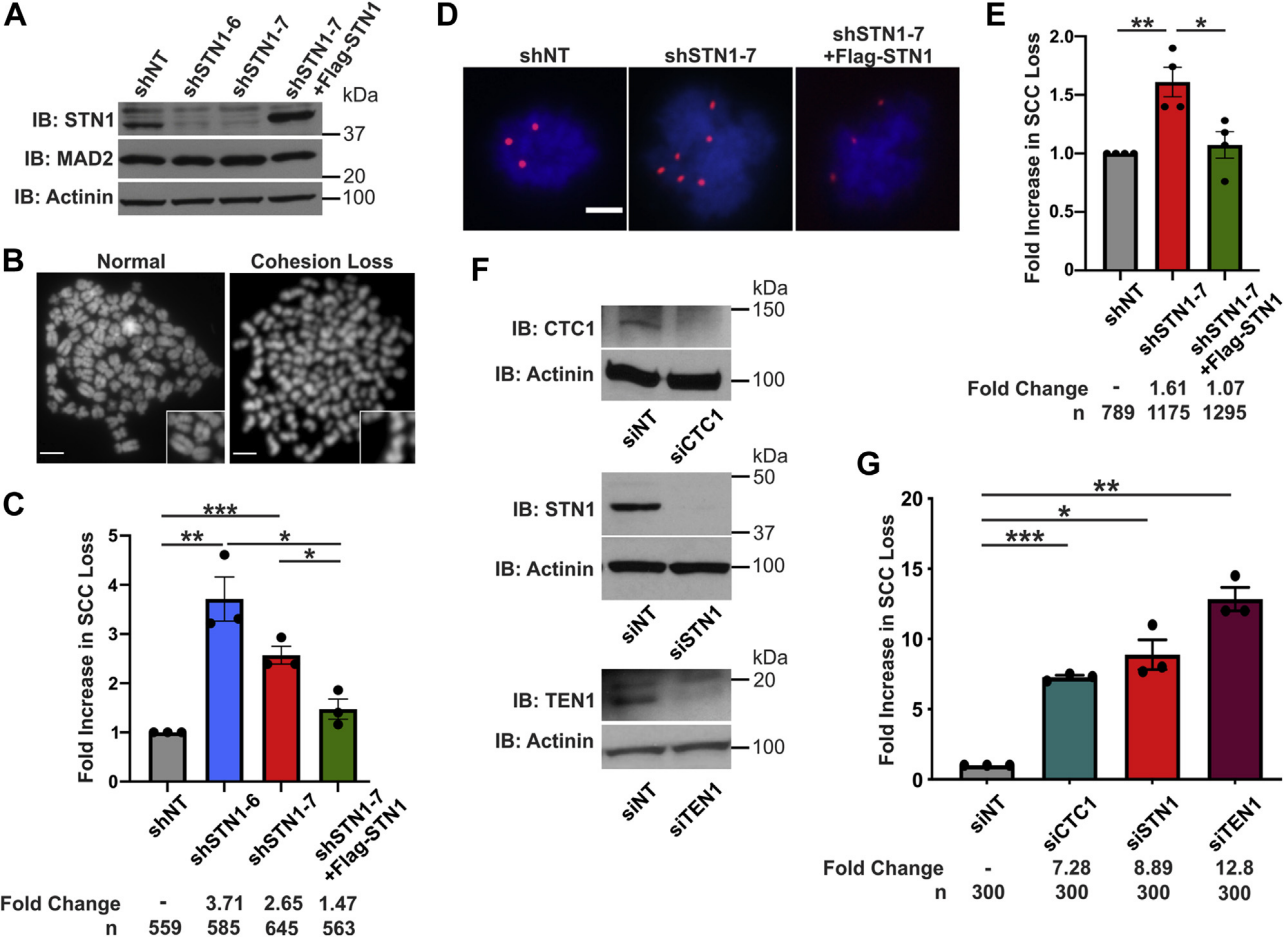
**CST deficiency results in SCC loss.***A*, Western blot of STN1 knockdown and MAD2 levels in HeLa cells. Actinin was used as the loading control. *B*, example images of metaphase spreads with normal chromosomes or SCC loss. The scale bar represents 5 μm. *C*, fold increase in cohesion loss after metaphase spread analysis. n = 3 independent, biological replicates. *D*, representative images of chromosome FISH from cells isolated by mitotic shake-off. *Red* represents centromere 6 probe; *blue* represents DAPI. The scale bar represents 5 μm. *E*, fold increase in nuclei with >4 chromosome 6 foci. n = 4 independent, biological replicates. *F*, knockdown of CTC1, STN1, or TEN1 by siRNA in HeLa cells. siNT was used as the nontarget control and actinin as the loading control. *G*, graph of SCC loss after metaphase spread analysis, as indicated. n = 3 independent, biological replicates. (∗*p* < 0.05, ∗∗*p* < 0.01, and ∗∗∗*p* < 0.001). CST, CTC1-STN1-TEN1; SCC, sister chromatid cohesion; shNT, nontargeting shRNA; shSTN1, shRNA knockdown of STN1; shSTN1+Flag-STN1, shSTN1-7 cells plus shRNA-resistant Flag-STN1.

While performing telomere-FISH in STN1 knockdown cells, we consistently observed metaphase spreads that had lost SCC. To determine whether depletion of STN1 increased cohesion loss, we quantified the number of metaphase spreads with SCC loss in HeLa cells with stable shRNA knockdown of STN1 (shSTN1) (Fig. 1, *A*–*C*) (16). Only metaphase spreads with at least 50% of the chromosomes having lost complete cohesion were scored as loss. By and large, these metaphase spreads had completely lost cohesion on all chromosomes, as shown in Figure 1*B*. In agreement with our observation, we observed a 2- to 4-fold increase in premature SCC loss in two separate shSTN1 clones, shSTN1-6 and shSTN1-7 (Fig. 1*C* and Fig. S1*C*). Furthermore, this increase was largely rescued by stable expression of a Flag-tagged shRNA-resistant STN1 construct in shSTN1-7 cells (shSTN1-7 +Flag-STN1). Previous studies suggest that a common off-target effect of RNA interference is MAD2 depletion (19). Loss of MAD2 also results in premature SCC loss. However, MAD2 levels were measured in the shSTN1 cells, and no significant changes were observed compared with controls (Fig. 1*A*).

To confirm our findings, we next performed mitotic shake-off to measure SCC loss after STN1 depletion. Processing of cells for standard metaphase spread analysis includes treatment with a hypotonic solution, which can release proteins from the chromatin (20). If cohesion is only partially lost, this can exacerbate SCC loss. To address this possibility, mitotic cells were collected, fixed, and spun onto slides without hypotonic treatment. Unlike standard metaphase spread preparation, chromosomes from cytospun metaphase cells are not clearly separated. Therefore, a chromosome-specific probe to centromere 6 was used to assess SCC loss by FISH, as previously described (21). Because HeLa cells are triploid for chromosome 6, three spots indicate the retention of SCC, whereas greater than three spots indicate loss. Consistent with metaphase spread analysis, knockdown of STN1 led to a significant increase in SCC loss (Fig. 1, *D* and *E* and Fig. S1*C*). However, the fold increase was less than that observed by standard metaphase spread analysis (Fig. 1*C*). This may be due to weakened but not complete SCC loss in a subset of cells or differences between the methodologies used.

Next, we determined whether cohesion loss was specific to STN1 depletion or due to a general CST loss. siRNA knockdown of CTC1, STN1, or TEN1 was performed followed by metaphase spread analysis (Fig. 1, *F* and *G* and Fig. S1*C*). Like shSTN1 cells, transient siRNA knockdown of individual CST subunits resulted in increased SCC loss. Finally, we confirmed that this phenotype was not cell-type specific by demonstrating SCC loss in HCT116 cells with conditional CTC1 KO (Fig. S1, *A* and *B*) and in both HCT116 and HEK293T cells with siRNA knockdown of STN1 (Fig. S1, *D*–*F* and *J*). Interestingly, the background percentage of loss in the HCT116 cells was significantly lower than in HeLa and HEK293T cells (Fig. S1, *E* and *F*). This is likely due to HCT116 cells having an intact p53 response (Fig. S1*G*) (22). In addition, CTC1 deletion (22) or STN1 depletion (Fig. S1*H*) in the HCT116 cells increased the number of G2/M, subG1, and aneuploid (>4n) cells, whereas no cell cycle defects were observed in HeLa (16) or HEK293T (Fig. S1*I*) cells after STN1 depletion. Like HeLa cells, HEK293T cells have a defective p53 response (Fig. S1*G*). However, regardless of p53 status, depletion of CST subunits increased SCC loss, suggesting that this phenotype is independent of p53. Together, these results indicate that CST promotes SCC.

### CST associates with the cohesin complex

**Figure 2:**
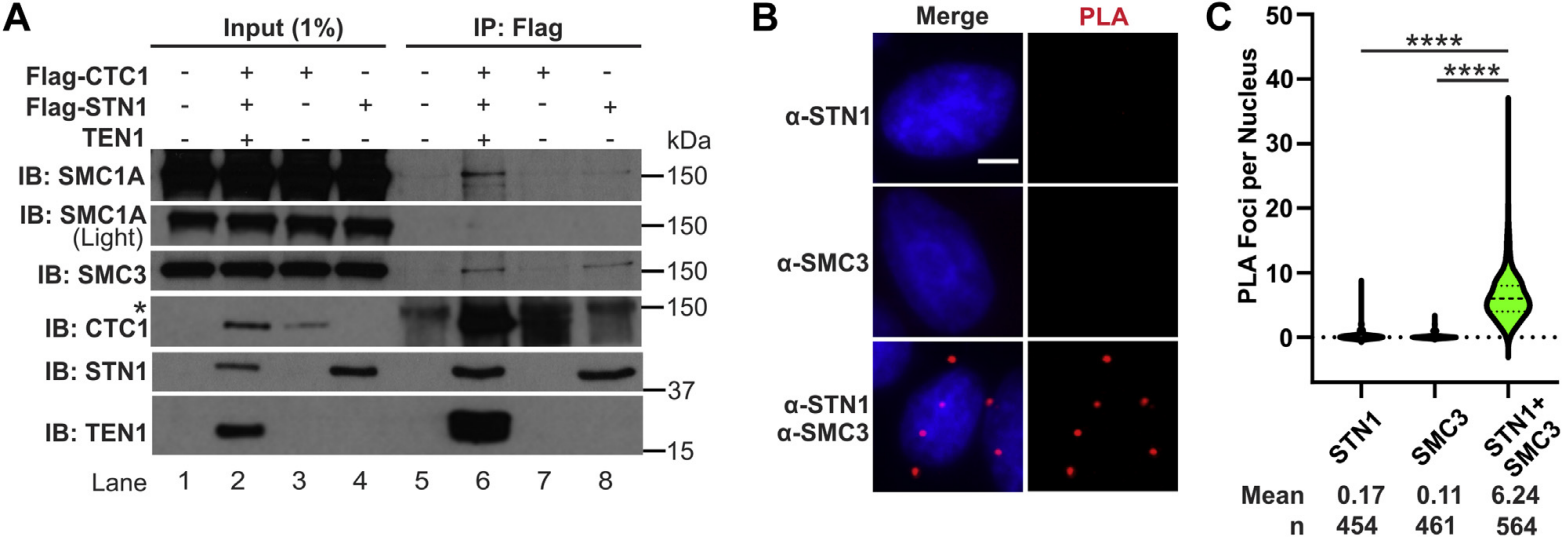
**CST associates with the cohesin complex.***A*, co-IP of nuclease-treated lysates from HEK293T cells expressing Flag-tagged CTC1, Flag-tagged STN1, or the full CST complex. The *asterisk* indicates background band. *B*, representative images of proximity ligation assay (PLA) performed in HeLa cells with antibodies to STN1 or SMC3 alone or in combination. *Red* represents PLA foci; *blue* represents DAPI. The scale bar represents 5 μm. *C*, violin plot of PLA foci per nucleus. Results are representative of four independent, biological experiments. *Bold dashed line* represents the median, and *dashed lines* represent the first and third quartiles. (∗∗∗∗*p* < 0.0001). co-IP, coimmunoprecipitation; CST, CTC1-STN1-TEN1.

We next addressed whether CST is associated with the cohesin complex by coimmunoprecipitation (co-IP) and proximity ligation assay (PLA) (Fig. 2). IP of epitope-tagged CST pulled down both endogenous SMC3 and SMC1A (Fig. 2*A*). To determine whether the association between cohesin and CST was through CTC1 or STN1, co-IP was performed with expression of Flag-CTC1 or Flag-STN1. STN1 alone pulled down SMC3, although at lower levels. Pulldown of CTC1 showed little to no association with SMC3 or SMC1A. This suggests that the entire CST complex, or at least multiple subunits, may be required for stable association with cohesin. As further confirmation of CST–cohesin association, we identified both SMC1A and SMC3 by MS after IP of Flag-STN1 in cells overexpressing all three CST subunits (Fig. S2) (Supplemental Data 1). Furthermore, PLA was performed using antibodies to endogenous STN1 and SMC3 to detect their association in cells (Fig. 2*B*) (23). PLA analysis showed ∼6 foci on average per cell compared with single antibody controls (Fig. 2*B*). For comparison, PLA with STN1 and known CST-interacting partners DNA polymerase α-primase and MCM2-7 showed 2.5 and 2.3 foci per cell on average, respectively, suggesting that CST–cohesin association is fairly robust and the number of PLA foci is likely an underrepresentation of their total colocalization in the cell (Fig. S3) (14, 17, 18, 24). Combined with the co-IP data, these findings reveal an unanticipated association between CST and the cohesin complex.

### CST is not required for SMC3 acetylation, cohesin loading, or mitotic progression

**Figure 3:**
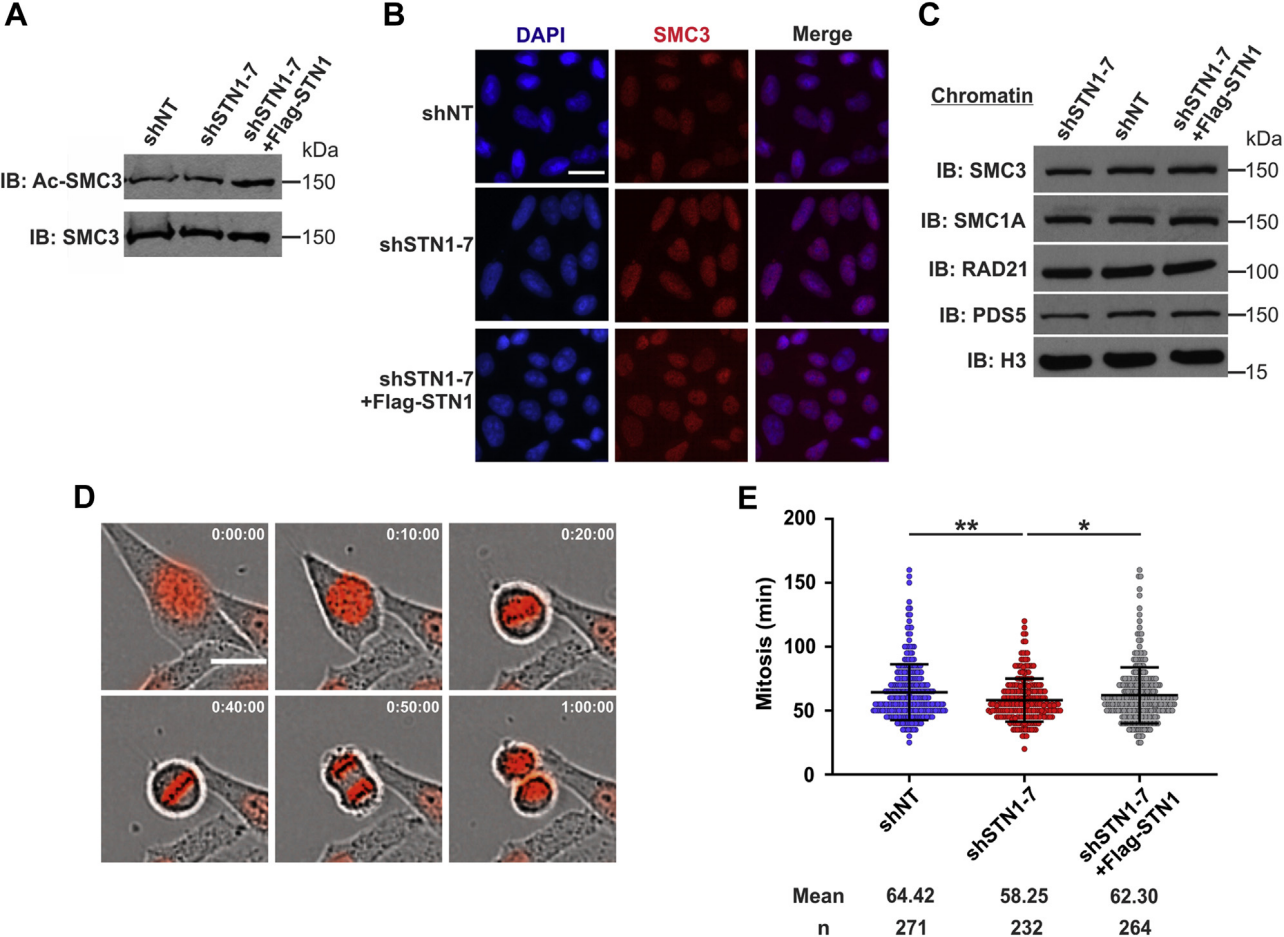
**STN1 depletion does not affect cohesin levels or mitotic timing.***A*, Western blot analysis of total SMC and acetylated SMC3 (Ac-SMC3) in HeLa cells, as indicated. *B*, representative images of SMC3 levels in pre-extracted cells. DAPI is shown in *blue* and SMC3 in *red*. The scale bar represents 20 μm. *C*, Western blot of chromatin-bound cohesin subunits. Histone H3 was used as the loading control. *D*, HeLa cells stably expressing H2B-RFP were imaged in 5-min intervals for 3 h. The scale bar represents 20 μm. *E*, dot plot of the time between nuclear envelope breakdown until cytokinesis in individual cells. *Black lines* and *numbers* below the graph indicate the average time in minutes to complete mitosis. (∗*p* < 0.05 and ∗∗*p* < 0.01).

A possible explanation for SCC loss after CST depletion is that it stabilizes cohesin loading or establishment (25, 26). Before genome duplication, cohesin binding is unstable and cohesin molecules quickly associate/dissociate from the DNA (27). As the genome is duplicated, cohesin is transferred from the unreplicated to the replicated sister chromatids and becomes stably bound. This process is partially facilitated by the acetylation of SMC3 (Ac-SMC3) (28, 29, 30, 31). Because CST aids in DNA replication, we tested whether Ac-SMC3 or the levels of total or chromatin-bound cohesin were decreased in STN1-depleted cells (Fig. 3, *A*–*C* and Fig. S4, *A* and *B*). However, we did not observe any changes in either Ac-SMC3 or chromatin-bound cohesin.

We next investigated whether mitotic progression was altered using live-cell imaging (Fig. 3, *D* and *E*). To visualize mitotic events, an H2B construct fused with red fluorescence protein was stably transduced into STN1-depleted and control cells (32). Cells were plated and imaged at 5-min intervals over a 3-h period. The time from prophase to the completion of cytokinesis was then measured as a readout of mitotic progression (Fig. 3*D*). Mitosis took approximately 1 h in the HeLa cells, consistent with previous studies (33). On average, shSTN1 cells took ∼5 min less to complete mitosis compared with control cells (Fig. 3*E*). Further breakdown of the timing from prophase to metaphase or metaphase to cytokinesis did not reveal any significant changes (Fig. S4*C*). While there was a slight decrease in overall timing of mitotic progression in shSTN1 cells, these changes are unlikely to explain the increase in SCC loss. Why premature loss of SCC does not lead to defects in mitotic progression in cells lacking CST is not entirely clear. However, work in *Drosophila* suggests that premature SCC loss is not always sufficient to trigger a robust spindle assembly checkpoint and that mitosis can still occur (34, 35). Moreover, recent work showed that various cancer cell lines continue to grow despite significant levels of cohesion loss (8, 26). Accordingly, mitotic progression and cell division may be unaffected despite increased SCC loss in STN1-depleted cells.

### CST promotes chromosome cohesion after replication stress

**Figure 4:**
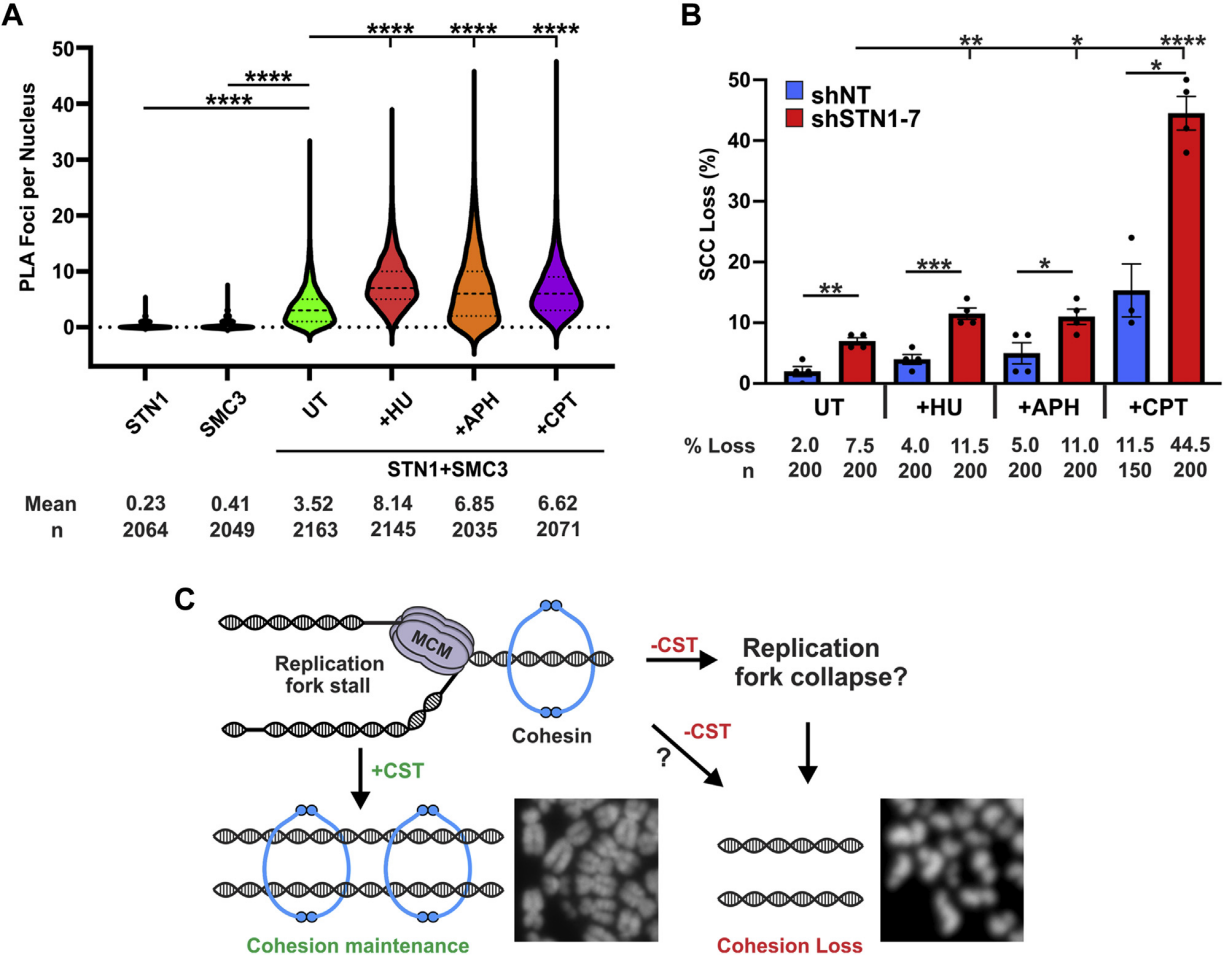
**Replication inhibition increases CST–cohesin association and SCC loss in STN1-depleted cells.***A*, violin plot of nuclear PLA foci in HeLa cells after treatment with DNA replication inhibitors. Treatment: hydroxyurea (HU) and aphidicolin (APH) for 2 h and camptothecin (CPT) for 1 h. The *bold dashed line* represents median and *dashed lines* the first and third quartiles. n = 3 independent, biological replicates. *B*, cohesion loss in HeLa shNT and shSTN1 cells after replication stress. Cells were treated with replication inhibitors as in panel *A* and then released for 8 h before metaphase spread preparation. Colcemid was added 2 h before collection. n = 3 independent, biological replicates. (∗*p* < 0.05, ∗∗*p* < 0.01, ∗∗∗*p* < 0.001, and ∗∗∗∗*p* < 0.0001). *C*, model of proposed CST function in cohesin maintenance. Images were cropped from those found in Figure 1*B*. CST, CTC1-STN1-TEN1; PLA, proximity ligation assay; SCC, sister chromatid cohesion; shSTN1, shRNA knockdown of STN1.

Because CST aids in DNA replication restart and SCC loss has been linked to replication stress, we sought to determine whether chemically induced replication stress would increase CST-cohesin association. Cells were treated with hydroxyurea (HU) or aphidicolin (APH) for 2 h or camptothecin (CPT) for 1 h. PLA was then performed with antibodies to endogenous STN1 and SMC3 (Fig. 4*A*). In all cases, we observed an ∼2-fold increase in PLA foci, indicating increased association after replication stress compared with untreated cells. This 2-fold increase is similar to increases observed in replication protein A-bound replication sites by PLA after HU treatment (36). This suggested to us that CST may prevent SCC loss after replication stress, so we tested SCC levels after treatment with replication inhibitors in shSTN1 cells. After treatment, cells were released into fresh media for 6 h, to allow cells in S phase to reach mitosis. They were then treated with colcemid, collected, and prepared for metaphase spread analysis (Fig. 4*B* and Fig. S5). In agreement with previous findings, SCC loss increased when cells were treated with replication inhibitors (8, 9). (CPT likely has the greatest effect because it is not reversible like HU and APH.) However, in the STN1-depleted cells, premature SCC loss was greatly increased above shNT cells, consistent with CST promoting SCC after replication stress.

## Discussion

In this study, we present data that CST is involved in chromosome cohesion, potentially through its association with the cohesin complex. Despite significant levels of SCC loss in CST-depleted cells, we did not detect defects in global cohesion levels, SMC3 acetylation, or mitotic progression. Instead, CST appears to be involved in SCC maintenance/remodeling at stalled replication forks. This idea is in line with recent work demonstrating that replication stress, caused by expression of oncogenes or treatment with DNA replication inhibitors, induces SCC loss in human cells (8, 9). Based on our data, we propose that CST acts at stalled replication forks to maintain or remodel chromosome cohesin after fork stalling or during fork restart (Fig. 4*C*). These findings highlight an unexpected function of CST in preserving genome integrity through the maintenance of SCC.

Over a decade ago, depletion of the replication fork stability factors Tipin/Tim and AND1 were shown to increase SCC loss, indirectly associating replication defects with chromatid cohesion (37, 38, 39, 40). Additional studies have implicated other DNA replication and repair factors in chromatid cohesion, and this past year, a pair of studies provided direct evidence that chemically induced replication stress leads to SCC loss in humans (8, 9). Both of these recent studies suggested that the cohesin antagonist WAPL is involved in cohesion maintenance after replication stress. Benedict *et al.* propose a model where WAPL removes cohesin to allow replication fork restart through RAD51-dependent mechanisms. Interestingly, CST is proposed to load RAD51 after fork stalling (15). Therefore, CST could be a key player in cohesin dynamics at stalled forks by facilitating fork restart and cohesion reestablishment. However, other studies suggest that cohesion is reinforced at stalled replication forks and double-strand breaks, in apparent contradiction to the previously mentioned studies (41). These studies, mostly performed in budding yeast, indicate that cohesin is recruited to stalled or collapsed replication forks for activation of the DNA damage response and homology-based fork restart. How this is reconciled with recent work, including this study, will require molecular characterization of the players involved. However, it likely depends on the fate of the replication fork after initial stalling and what happens to cohesin after the “repair” event. It is also possible that cohesin dynamics at stalled replication forks differs in humans compared with lower eukaryotes, although evidence of cohesion reinforcement has also been observed in human cells after ionizing radiation (42).

Another potential link between CST and cohesion maintenance is that CST interacts with both MCM2-7 and AND-1 (14). Recent work identified interaction between the MCM2-7 helicase and ESCO2 as necessary for cohesion establishment (25, 28, 31). As mentioned above, AND-1 is involved in replication fork stability and helps maintain SCC (39). Studies in yeast suggest that AND-1 (known as Ctf4 in yeast) stabilizes critical interactions between replisome components and the Chl1 helicase, which is involved in chromatid cohesion (43, 44). We previously showed that loss of CST leads to decreased chromatin-bound AND-1, which could provide an explanation for increased SCC loss in the absence of CST. CST could also directly interact with cohesin to promote cohesin remodeling or reestablishment. However, our results have not definitively shown whether CST directly interacts with cohesin or is associated with it *via* interactions with components of the replisome, such as MCM2-7. Future work is needed to define how specific interactions at the replication fork affect chromosome cohesion and fork restart/protection after stalling.

While the function of CST in replication restart/rescue is still unclear, CST is not a general replication factor but rather plays a specialized role in facilitating replication through G-rich regions of the genome (*e.g.*, telomeres, CpG islands) (13, 15, 16). Because CST promotes replication at specific sites, one might predict that the absence of CST would cause partial *versus* total loss of chromosome cohesion, as has been previously observed with depletion of the cohesin subunits SA1 or SA2 (21). Instead, CST depletion leads to complete SCC loss by metaphase spread analysis and mitotic shake-off (Fig. 1). These findings demonstrate that SCC loss is not restricted to telomeres or specific regions of the genome but instead a complete breakdown of SCC. The reason for complete cohesion loss remains unclear. However, recent studies suggest that complete cohesion loss is common across a variety of cancer cells, which seems to have little effect on cellular division (8). It is possible that the gradual accumulation of SCC loss due to replication stress or excessive DNA damage triggers genome-wide cohesin unloading through an unknown mechanism. Perhaps such a pathway is used to induce cell death and prevent the propagation of cells with high levels of genome instability induced by replication stress. In cancer, such pathways could be disengaged to allow cell division and aneuploidy, despite SCC loss. However, future studies are required to fully investigate the connection between replication stress, SCC, and aneuploidy.

## Experimental procedures

### Cell culture

HeLa 1.2.11 cells were cultured in RPMI 1640 media, HEK293T in DMEM, and HCT116 in McCoy’s 5A media supplemented with 10% fetal bovine serum and 1% penicillin/streptomycin at 37 °C with 5% CO_2_. Stable HeLa 1.2.11 shRNA knockdown and HCT116 CTC1 inducible KO lines have been previously described (16, 45). Cell lines were regularly checked for *Mycoplasma* contamination. siRNA experiments were performed using 25 nM ON-TARGETplus siRNA SMARTpool (Dharmacon) to CTC1 (L-014585-01), STN1 (L-016208-02), TEN1 (L-187549-00), or nontargeting control (D-00180-10). siRNAs were transfected into cells with Lipofectamine RNAiMAX (Thermo Fisher Scientific) for 72 h before collection.

### Metaphase spreads

Metaphase spreads were prepared as previously described (46) and then stained for 8 to 10 min with Giemsa stain (Ricca Chemical). SCC loss was counted when at least half of the chromosomes had lost complete cohesion. In most cases, the entire metaphase spread had lost complete cohesion.

### Whole-cell lysate, chromatin fractionation, and Western blot analysis

These techniques were performed, as previously described (14).

### Antibodies and chemical inhibitors

#### Primary

The primary antibodies used were as follows: SMC1A (Bethyl Laboratories, A300-055A), SMC3 (Bethyl Laboratories, A300-060A), acetylated SMC3 (kindly provided by Dr Prasad Jallepalli), RAD21 (SCC1) (Bethyl Laboratories, A300-080A), SCC-112 (PDS5) (Bethyl Laboratories, A300-089A), OBFC1 (STN1) (Novus Biologicals, NBP2-01006), CTC1 (45), α-tubulin (MilliporeSigma, T-9026), TEN1 (47), MAD2 (Bethyl Laboratories, A300-301A), α-actinin (Santa Cruz Biotechnology, SC17829), H3 (Cell Signaling Technology, 9715), Flag (Thermo Fisher Scientific, MA1-91878, PA1-984B), PolA1 (Bethyl Laboratories, A302-805A), MCM7 (Santa Cruz Biotechnology, sc22782), p53 (Cell Signaling Technology, 2524S), and p21 (Santa Cruz Biotechnology, sc-6246).

#### Secondary

The secondary antibodies used were from Thermo Fisher Scientific: anti-rabbit-HRP (32460), anti-mouse-HRP (32430), goat anti-rabbit Alexa Fluor 594 (A-11037).

#### Chemical inhibitors

Cells were treated with APH (1 μM, MilliporeSigma, 178273) or HU (2 mM, MilliporeSigma, 400046) for 2 h or (S)-(+)-CPT (1 μM, MilliporeSigma, C9911) for 1 h.

### Live-cell imaging

MSCV-H2B-mRFP1 was created by replacing GFP in MSCV-GFP with the H2B-mRFP1 from pCS-H2B-mRFP1, using NotI and XhoI. pCS-H2B-mRFP1 was a gift from Dr Sean Megason (Addgene #53745) and MSCV-GFP a gift from Dr Tannishtha Reya (Addgene #20672). HeLa cells were transduced with retrovirus produced in HEK293T, and RFP-positive cells were selected through two rounds of fluorescence-activated cell sorting. Approximately 3000 cells were plated into 96-well Incucyte ImageLock plates 24 h before imaging. Cells were then imaged at 5-min intervals for 3 h under a 20× objective, using the Incucyte S3 Live-Cell Analysis System.

### FISH

After collection, cells were washed with PBS and fixed by dropwise addition of Carnoy’s solution (3:1 methanol:acetic acid) with gentle vortexing. Cells were incubated on ice for 10 min and spun down, and the supernatant was removed. Fresh Carnoy’s solution was then added and cells stored at 4 °C. Before cytospin, cells were counted and resuspended in fresh Carnoy’s solution to a concentration of approximately 500 cells/μl. One hundred microliter of cell suspension was then spun onto slides at 10,000 rpm for 2 min using 3-well adaptors (StatSpin CytoFuge 2). Slides were washed with Carnoy’s solution and allowed to dry. Chromosome-specific FISH was then performed with a Texas Red-labeled chromosome 6 alpha satellite probe, following the manufacturer’s protocol (Cytocell).

### Immunofluorescence

Cells were pre-extracted with ice-cold 1× CSK buffer (10 mM Hepes, pH 7.4, 0.3 M sucrose, 100 mM NaCl, 3 mM MgCl_2_) containing 0.1% Triton X-100 for 2 min at room temperature (RT) and then fixed with ice-cold 100% methanol at −20 °C for 10 min. Immunofluorescence was then performed as previously described (14).

### co-IP

Co-IP was performed as previously described (14). Plasmids used include pcDNA3.1-Flag-CTC1, pcDNA3.1-Flag-STN1, and pCMV6-TEN1 (14, 16).

### PLA

HeLa 1.2.11 cells were fixed for 20 min at RT with 4% formaldehyde in 1× PBS followed by permeabilization with 100% methanol for 20 min at −20 °C. Subsequent steps were performed with the Duolink PLA kit (MilliporeSigma) as previously described (23), except the first wash after primary incubation was performed using wash buffer A, not 5% BSA in 1× PBS. The following primary antibodies were used: 1:100 mouse α-STN1, 1:600 rabbit α-SMC3, 1:100 rabbit α-PolA1, and 1:500 rabbit α-MCM7.

### Image analysis and statistics

For immunofluorescence, FISH, and PLA, images were taken on an EVOS FL microscope, using a 40× or 60× objective (Thermo Fisher Scientific). At minimum, five images were scored per independent, biological experiment for each condition. Image analysis (Figs. 2*B* and 3*B* and Figs. S3 and S4) was performed with CellProfiler. Error bars indicate the ±SEM of at least three independent biological experiments. All *p*-values were calculated by an unpaired, two-tailed *t* test.

## Data availability

The mass spectrometry proteomics data have been deposited to the ProteomeXchange Consortium *via* the PRIDE (48) partner repository with the dataset identifier PXD026264.

## Supporting information

This article contains supporting information (22, 49, 50).

## Conflict of interest

The authors declare that they have no conflicts of interest with the contents of this article.
