# Supplementary material for: The DNA-binding protein CST associates with the cohesin complex and promotes chromosome cohesion
Source: J Biol Chem. 2021 Jul 30;297(3):101026. doi: 10.1016/j.jbc.2021.101026 (PMC8390553; doi:10.1016/j.jbc.2021.101026)
Supplement: Supplemental Figures S1–S5 [file mmc1.pdf]

# SUPPORTING INFORMATION

# The DNA-binding protein CST associates with the cohesin complex and promotes sister chromosome cohesion

**P. Logan Schuck[1], Lauren E. Ball[2] and Jason A. Stewart[1]***

From the [1]Department of Biological Sciences, University of South Carolina, Columbia, SC, USA; [2]Department of Cell and Molecular Pharmacology, Medical University of South Carolina, Charleston, SC, USA

*Corresponding author: jason.stewart@sc.edu

**Supporting Experimental Procedures**

*Flow Cytometry*

Samples were prepared as previously described (22). Gating and analysis were performed using FlowJo.

*Mass spectrometry*

Sample preparation: Three replicate affinity-purifications were performed for CST or a negative control for non-specific binding. Eluates were separated by SDS-PAGE, Coomassie stained, and each lane was cut into three bands. Gel pieces were de-stained and proteins were reduced in 10 mM dithiothreitol (Thermo Scientific, Rockford, IL) at 55°C, and alkylated in 25 mM iodoacetamide (Thermo) for 30 minutes at room temperature in the dark. The protein was digested with trypsin (Sigma) (100 ng) overnight at 37°C. Digestion was quenched by the addition of trifluoroacetic acid (TFA) to a final concentration of 1%, and peptides were extracted from the gel and dried.

LC-MS/MS: Peptides were loaded onto a trap column and separated with a 75 µm x 30 cm analytical column (packed in house, C18-Reprosil-AQ Pur RP 1.9 µm particles, Dr. Maisch, GmbH) at 60°C using a gradient from 5% B to 40% B in 180 min (Solvent A: 0.2% formic acid in 2% acetonitrile; Solvent B: 2% formic acid in 98% acetonitrile) on a U3000 nano LC system. The flow rate was 180 nl/min. Mass spectra were acquired on an Orbitrap Elite (Thermo Scientific) in data dependent mode with one FTMS survey scan, mass range of m/z 400-1700 Th, followed by collisional dissociation of the ten most intense ions and detection in the ion trap. The automatic gain control target value was $10^{6}$ ions for the survey MS scan, the resolution was 60,000 at m/z 400 Th. Ions with a +1 charge were excluded from selection. Dynamic exclusion was enabled with a repeat count of 1, duration of 30 sec, exclusion list size of 50, and exclusion duration of 180 sec. Data were only acquired during the gradient omitting the wash and recalibration time. Chromatography mode was enabled with an expected peak width of 30 sec and a minimum threshold of 1000. Three blanks were run between each sample to avoid carry over.

Protein Identification: Data were searched using MaxQuant v.1.6.1.5 against a Human Uniprot protein database including common contaminants. The false discovery rate, determined using a reversed database strategy, was set at 0.01 at the protein and peptide level. Fully tryptic peptides with a minimum of 7 residues were required including cleavage between lysine and proline. Two missed cleavages and two modifications were permitted. LC-MS/MS analyses were performed in triplicate for each condition and searched together matching between runs with a 0.7 min window. The fast LFQ feature was disabled. The first search was performed with a 25 ppm mass tolerance, after recalibration a 4.5 ppm tolerance was used for the main search. At least two peptides were required for protein quantification with at least one unique peptide. Parameters included static modification of cysteine with carbamidomethyl, variable

N-terminal acetylation, and methionine oxidation. Intensity measurements were quantified and normalized by the MaxQuant LFQ algorithm (49).

Data processing: The protein groups text file was processed in Perseus (Version 1.6.2.1) (50) and matches to the reversed database, contaminants, and proteins only identified by modified peptides were removed. Identified proteins were required to have at least two peptides with at least one unique peptide. The normalized LFQ intensities were log2 transformed. The data were filtered to retain proteins quantified in one of the three biological replicates of the experimental immunoprecipitation. Missing values were replaced by random values imputed from a normal distribution using a width of 0.6, downshift of 1.5, and the total matrix of 3 control and 3 experimental immunoprecipitations. The mean log2 protein intensities were compared between the CST IP and control IP using a Student's t-test. Log2 fold changes in protein intensities and the –log10 pval are shown in the volcano plot.

REFERENCES:

22. Ackerson, S.M., Gable, C.I., and Stewart, J.A. (2020) Human CTC1 promotes TopBP1 stability and CHK1 phosphorylation in response to telomere dysfunction and global replication stress. *Cell Cycle* **19**, 3491-3507

49. Cox, J., Hein, M.Y., Luber, C.A., Paron, I., Nagaraj, N., and Mann, M. (2014) Accurate proteome-wide label-free quantification by delayed normalization and maximal peptide ratio extraction, termed MaxLFQ. *Mol Cell Proteomics.* **13**, 2513-26

50. Tyanova, S., Temu, T., Sinitcyn, P., Carlson, A., Hein, M.Y., Geiger, T., Mann, M., and Cox, J. (2016) The Perseus computational platform for comprehensive analysis of (prote)omics data. *Nat Methods.* **13**, 731-40.

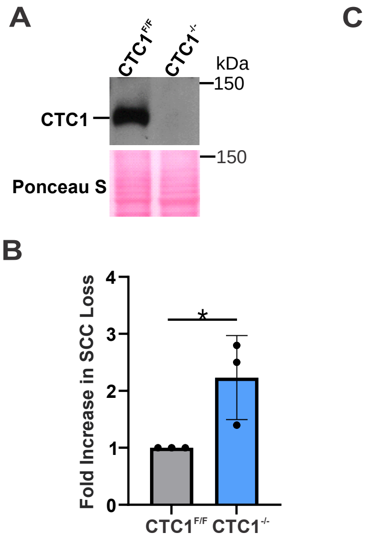


C

| Spreads | Exp 1 | Exp 2 | Exp 3 | Total |
|---|---|---|---|---|
| **HeLa** | | | | |
| shNT | 8/167 (4.8%) | 21/187 (11.2%) | 31/205 (15.1%) | 60/559 (10.7%) |
| shSTN1-6 | 39/175 (22.3%) | 76/210 (36.1%) | 100/200 (50%) | 215/585 (36.8%) |
| shSTN1-7 | 30/239 (12.5%) | 55/205 (26.8%) | 89/201 (44.2%) | 174/645 (27.0%) |
| shSTN1-7 +Flag-STN1 | 7/150 (4.7%) | 26/200 (13.0%) | 60/213 (28.2%) | 93/563 (16.5%) |
| **HeLa** | | | | |
| siNT | 3/100 (3.0%) | 3/100 (3.0%) | 2/100 (2.0%) | 8/300 (2.7%) |
| siCTC1 | 22/100 (22.0%) | 21/100 (21.0%) | 23/100 (23.0%) | 66/300 (22.0%) |
| siSTN1 | 24/100 (24.0%) | 23/100 (23.0%) | 22/100 (22.0%) | 69/300 (23%) |
| siTEN1 | 36/100 (36.0%) | 36/100 (36.0%) | 29/100 (29.0%) | 101/300 (33.7%) |
| **HCT116** | | | | |
| CTC1 F/F | 2/150 (1.3%) | 5/200 (2.5%) | 5/200 (2.5%) | 12/550 (2.2%) |
| CTC1 -/- | 5/150 (3.3%) | 7/200 (3.5%) | 14/200 (7.0%) | 26/550 (4.7%) |

*SCC loss over the total number of metaphases scored. Percentage SCC loss in parenthesis.

| FISH | Exp 1 | Exp 2 | Exp 3 | Exp 4 | Total |
|---|---|---|---|---|---|
| **HeLa** | | | | | |
| shNT | 25/159 (15.7%) | 29/275 (10.6%) | 25/156 (16.0%) | 38/199 (19.1%) | 117/789 (14.8%) |
| shSTN1 | 47/213 (22.1%) | 86/581 (14.8%) | 58/211 (27.5%) | 62/170 (36.5%) | 253/1175 (21.5%) |
| shSTN1-7 +Flag-STN1 | 27/145 (18.6%) | 57/709 (8.0%) | 45/220 (20.5%) | 54/221 (24.4%) | 183/1295 (14.1%) |

*SCC loss over the total number of metaphases scored. Percentage SCC loss in parenthesis.

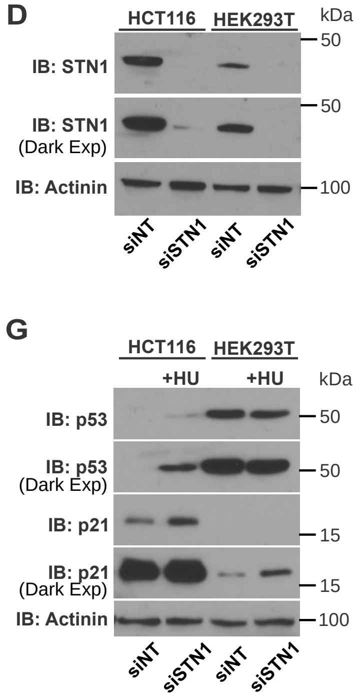


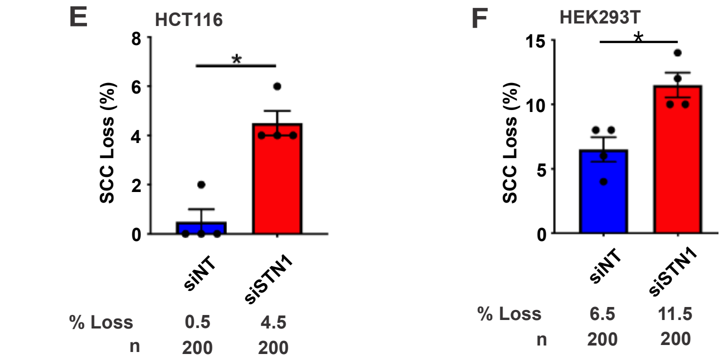


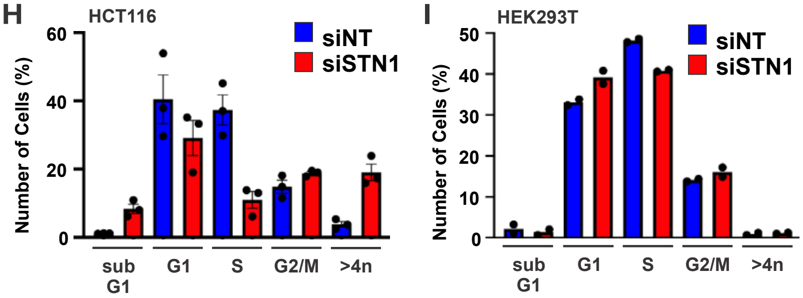


J

| | Exp 1 | Exp 2 | Exp 3 | Exp 4 | Total |
|---|---|---|---|---|---|
| **HCT116** | | | | | |
| siNT | 1/50 (2%) | 0/50 (0%) | 0/50 (0%) | 0/50 (0%) | 1/200 (0.5%) |
| siSTN1 | 2/50 (4%) | 3/50 (6%) | 2/50 (4%) | 2/50 (4%) | 9/200 (4.5%) |
| **HEK 293T** | | | | | |
| siNT | 2/50 (4%) | 4/50 (8%) | 3/50 (6%) | 4/50 (8%) | 13/200 (6.5%) |
| siSTN1 | 5/50 (10%) | 7/50 (14%) | 6/50 (12%) | 5/50 (10%) | 23/200 (11.5%) |

*SCC loss over the total number of metaphases scored. Percentage SCC loss in parenthesis.

**Figure S1. Supporting information related to Figure 1**. (A) Western blot of CTC1 in HCT116 cells. Conditional gene disruption of CTC1$^{F/F}$ was induced by the addition of tamoxifen (TAM) to initiate Cre-induced gene disruption (CTC1$^{-/-}$). Ponceau S: loading control. (B) Metaphase spread analysis of SCC loss in HCT116 cells, as indicated. Samples were collected on day 15 after TAM addition. Replicates

were standardized to CTC1$^{F/F}$ cells. (*P < 0.05). (C) Raw numbers and percentages of SCC loss from individual trials in Fig. 1 and S1B. Spreads refers to metaphase spread analysis in Fig. 1C, 1G and S1B, as indicated. FISH refers to analysis of the chromosome specific FISH experiments in Fig. 1E. (D) Western blot of STN1 knockdown by siRNA in HCT116 or HEK293T cells. siNT: non-target control, Actinin: loading control. (E-F) Percent of cohesion loss in HCT116 (E) or HEK293T (F) cells following metaphase spread analysis, as indicated. n=4 independent, biological replicates. (G) Western blot of p53 and p21 levels. +HU: 2mM HU treatment for 24 h. Dark Exp: extended exposure time. Actinin: loading control. (H-I) Percentage of cells in different phases of the cell cycle in HCT116 (H) or HEK293T cells (I) cells. (J) Raw numbers and percentages of SCC loss from individual trials in Fig. S1E-F. (*P < 0.05)

**A**

Total Number of Peptides

| | Trial 1 | | Trial 2 | | Trial 3 | |
|---|---|---|---|---|---|---|
| | Mock | CST IP | Mock | CST IP | Mock | CST IP |
| STN1 | 14 | 446 | 1 | 738 | 12 | 629 |
| CTC1 | 1 | 30 | 0 | 41 | 1 | 47 |
| TEN1 | 0 | 3 | 0 | 4 | 0 | 4 |
| POLA1 | 3 | 19 | 1 | 23 | 0 | 22 |
| POLA2 | 0 | 12 | 0 | 25 | 0 | 22 |
| PRIM1 | 0 | 7 | 0 | 4 | 0 | 9 |
| SMC1A | 0 | 5 | 0 | 2 | 1 | 3 |
| SMC3 | 0 | 3 | 1 | 3 | 1 | 3 |

**B**

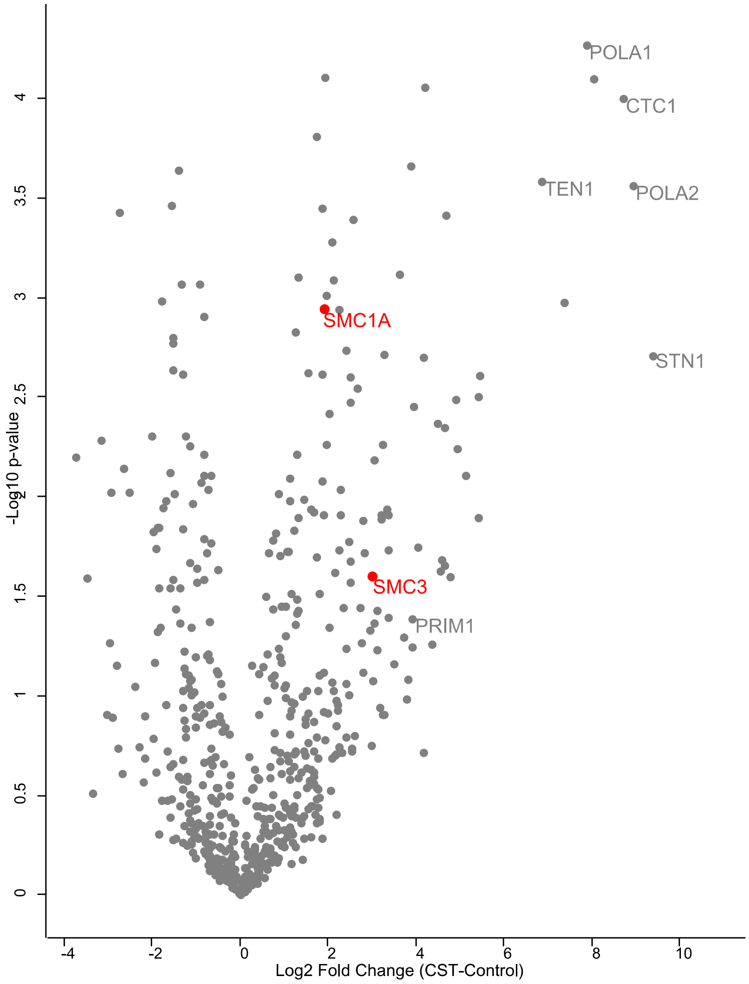


**Figure S2. CST interacts with the cohesin complex.** (A) Total number of peptides identified in three independent replicates of mass spectrometry to identify CST interacting partners. All three CST subunits were overexpressed in HEK 293T cells. Flag-tagged STN1 was pulled down and samples sent for analysis by mass spectrometry. (B) Volcano plot showing the log2 fold changes in protein intensity from CST immunoprecipitation as compared to control versus the log10 Student's t-test p value.

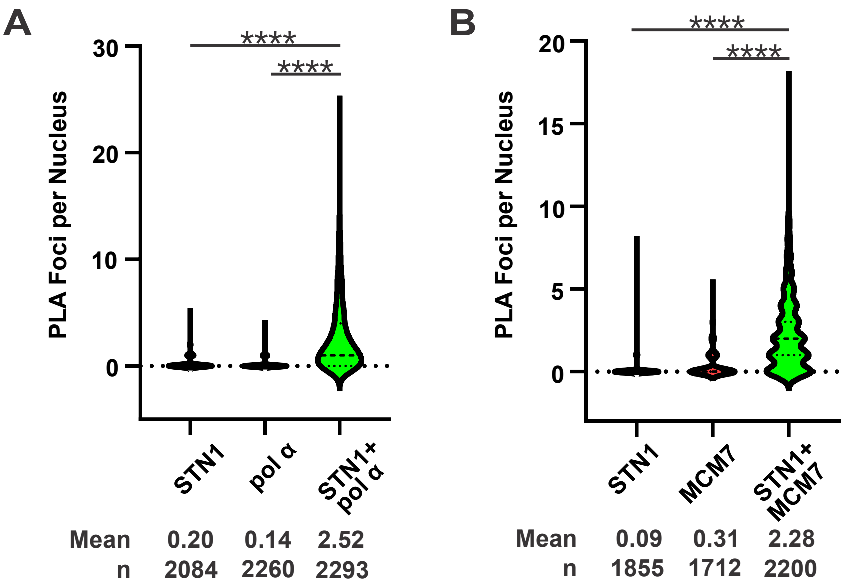


**Figure S3. PLA demonstrating STN1 interaction with pol α and MCM2-7**. (A) Proximity ligation assay (PLA) performed in HeLa cells with antibodies to STN1, the pol α subunit PolA1 or in combination (STN1+ pol α). (B) PLA was performed in HeLa cells as in (A) with antibodies to STN1 and MCM7 (STN1+MCM7). Violin plots of PLA foci per nucleus. Results for (A) and (B) are representative of two independent, biological experiments. Bold dashed line: median, dashed lines: first and third quartiles. (****$P < 0.0001$).

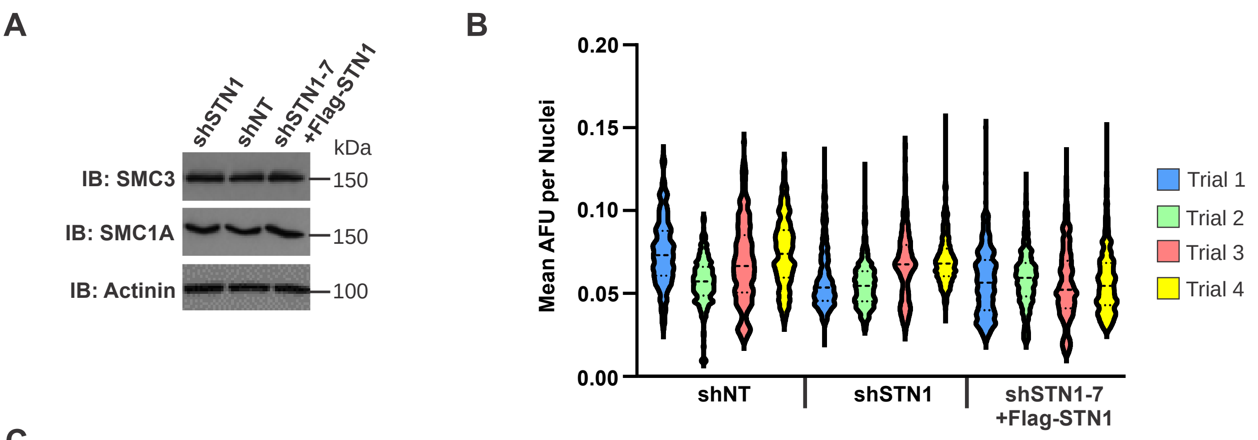


C

**Prophase to Metaphase**

| | Trial 1 | Trial 2 | Trial 3 | Average | SD |
|---|---|---|---|---|---|
| shSTN1 | 0:17:12 | 0:18:24 | 0:23:54 | 0:19:50 | 0:03:34 |
| shNT | 0:19:15 | 0:17:48 | 0:30:40 | 0:22:34 | 0:07:03 |
| Res | 0:14:46 | 0:16:34 | 0:24:53 | 0:18:44 | 0:05:24 |

**Metaphase to Cytokinesis**

| | Trial 1 | Trial 2 | Trial 3 | Average | SD |
|---|---|---|---|---|---|
| shSTN1 | 0:42:39 | 0:42:59 | 0:33:34 | 0:39:44 | 0:05:21 |
| shNT | 0:34:28 | 0:39:24 | 0:40:30 | 0:38:07 | 0:03:13 |
| Res | 0:38:22 | 0:46:58 | 0:39:45 | 0:41:42 | 0:04:37 |

**Figure S4. CST deficiency does not affect cohesin levels or mitotic timing.** (A) Western blot analysis of total cohesin levels in HeLa shSTN1 knockdown cells using whole cell extracts. Actinin serves as a loading control. (B) Nuclear SMC3 immunofluorescence intensity was measured in 4 independent trials (see Fig 3B). Violin plots of each trial, which shows no significant changes in SMC3 levels in shSTN1 cells versus controls. (C) Top, time between nuclear envelope breakdown to metaphase plate formation was measured in HeLa shSTN1 cells expressing H2B-RFP (see Fig 3D). Bottom, time between metaphase plate formation to cytokinesis was measured for each mitotic cell. n=3 independent biological experiments. SD: standard deviation. Time represented in hours: minutes: seconds.

| | Exp 1 | Exp 2 | Exp 3 | Exp 4 | Total |
|---|---|---|---|---|---|
| **HeLa** | | | | | |
| shNT | 2/50 (4%) | 0/50 (0%) | 1/50 (2%) | 1/50 (2%) | 4/200 (2%) |
| shSTN1 | 4/50 (8%) | 3/50 (6%) | 4/50 (8%) | 4/50 (8%) | 15/200 (7.5%) |
| shNT +HU | 2/50 (4%) | 2/50 (4%) | 1/50 (2%) | 3/50 (6%) | 8/200 (4%) |
| shSTN1 +HU | 5/50 (10%) | 7/50 (14%) | 5/50 (10%) | 6/50 (12%) | 23/200 (11.5%) |
| shNT +APH | 4/50 (8%) | 4/50 (8%) | 1/50 (2%) | 1/50 (2%) | 10/200 (5%) |
| shSTN1 +APH | 7/50 (14%) | 6/50 (12%) | 5/50 (10%) | 4/50 (8%) | 22/200 (11%) |
| shNT +CPT | 12/50 (24%) | 5/50 (10%) | | 6/50 (12%) | 23/150 (11.5%) |
| shSTN1 +CPT | 21/50 (42%) | 25/50 (50%) | 19/50 (38%) | 24/50 (48%) | 89/200 (44.5%) |

**Figure S5. Supporting information for Figure 4B.** Raw numbers and percentages of SCC loss from individual trials. Exp 3 shNT +CPT was not included due to technical issues that led to low cells counts and an insufficient numbers of metaphase spreads for analysis.
